# Cognitive Empathy as Imagination: Evidence From Reading the Mind in the Eyes in Autism and Schizotypy

**DOI:** 10.3389/fpsyt.2021.665721

**Published:** 2021-04-01

**Authors:** Priya Nahal, Peter L. Hurd, Silven Read, Bernard Crespi

**Affiliations:** ^1^Department of Biological Sciences, Simon Fraser University, Burnaby, BC, Canada; ^2^Centre for Neuroscience, Department of Psychology, University of Alberta, Edmonton, AB, Canada

**Keywords:** empathy, autism, schizotypy, RMET, imagination, sociality

## Abstract

How is cognitive empathy related to sociality, imagination, and other psychological constructs? How is it altered in disorders of human social cognition? We leveraged a large data set (1,168 students, 62% female) on the Reading the Mind in the Eyes test (RMET), the Autism Quotient (AQ), and the Schizotypal Personality Questionnaire (SPQ-BR) to test the hypotheses that the RMET, as a metric of cognitive empathy, reflects mainly social abilities, imagination, or both. RMET showed the expected female bias in performance, though only for eyes that expressed emotions and not for neutral expressions. RMET performance was significantly, and more strongly, associated with the AQ and SPQ subscales that reflect aspects of imagination (AQ-Imagination and SPQ-Magical Ideation) than aspects of social abilities (AQ-Social, AQ-Communication, and SPQ-Interpersonal subscales). These results were confirmed with multiple regression analysis, which also implicated increased attention (AQ-Attention Switching and, marginally non-significantly, AQ-Attention to Detail) in RMET performance. The two imagination-related correlates of RMET performance also show the strongest sex biases for the AQ and SPQ: male biased in AQ-Imagination, and female biased in SPQ-Magical Ideation, with small to medium effect sizes. Taken together, these findings suggest that cognitive empathy, as quantified by the RMET, centrally involves imagination, which is underdeveloped (with a male bias) on the autism spectrum and overdeveloped (with a female bias) on the schizotypy spectrum, with optimal emotion-recognition performance intermediate between the two. The results, in conjunction with previous studies, implicate a combination of optimal imagination and focused attention in enhanced RMET performance.

## Introduction

Cognitive empathy centrally involves the recognition in others of emotions, beliefs, and intentions. Such recognition of mental states derives in part from visual cues, especially those involving the eye region of the face, which is highly expressive due to its finely controlled musculature and variation in iris and pupil positions relative to the white sclera (1). Human social interactions thus typically comprise rapid, fluid, and complex changes in eye-region facial cues that convey information about emotions and cognitive states.

Abilities to interpret and generate eye region cues and other facial cues of emotion and cognition vary notably among individuals, and, when sufficiently altered from biological and cultural norms, generate problems in social interaction and communication. At extremes, such problems manifest as so-called disorders of social cognition. Most psychiatric disorders involve some degree and form of social problems, given the highly social nature of human psychology (2). However, autism spectrum disorders and psychotic-affective spectrum disorders (mainly schizophrenia, bipolar disorder, depression, their less-severe dimensional expressions, and highly-comorbid conditions such as borderline personality) present most specifically and intensely with alterations to social cognition and emotion. As such, these disorders have been studied especially intensely with regard to cognitive empathy and the psychological tests that quantify and characterize it.

Most psychological studies of cognitive empathy have analyzed this construct at the level of subjects with psychiatric diagnoses compared to controls. Deficits are almost always found, but limited insights can be derived from their presence and strength. These limitations arise because clinical frameworks for investigation are inherently constrained by the high neurological and psychological heterogeneity of symptom expression found within each disorder (3, 4), by the general cognitive deficits, and effects of medication, that can alter results in unpredictable ways, and by the great variety of ways that social cognition can become impaired. One approach to surmounting these limitations is to focus on specific symptoms of disorders rather than dichotomous diagnoses, and to do so in non-clinical populations that express disorder-related phenotypes in much less extreme forms.

In this study, we used a paradigmatic test for cognitive empathic abilities, the Reading the Mind in the Eyes Test (RMET) (5), in a non-clinical population of subjects who were quantified for the different dimensions of autism spectrum and schizotypy spectrum psychological traits. Our main goal was to determine what aspects of autistic and schizotypy spectrum cognition are associated with RMET performance, in the broader contexts of how autism and schizotypy are related to one another, and how they are associated with sex. In this general framework, higher autism spectrum traits can be predicted to be associated with lower RMET scores due to under-mentalizing, and higher positively-schizotypal traits should be associated with lower RMET scores due to over-mentalizing (6, 7). Here, under-mentalizing refers to a lack or reduction in attribution of agency, intention, feelings and other mental states to others, and over-mentalizing refers to relatively increased and complex attributions of agency, intentions, feelings and other mental states to others that are unsupportable from the information objectively available. Previous work has not addressed the question of how and why autism-related traits and schizotypy-related traits affect RMET performance, using the same non-clinical population.

The RMET involves choosing which of four words corresponds to the emotion or mental state displayed by a person, from a rectangular photograph of the eye region of their face. To relate RMET to autism in this study, autism spectrum traits were quantified using the Autism Quotient, a self-report test with five subscales that correspond to primary symptom dimensions of autism (8). Schizotypy spectrum traits were quantified using the Schizotypal Personality Questionnaire – Brief Revised (SPQ), a self-report test with seven subscales that quantify the main dimensions of schizotypy (9). RMET, AQ, and SPQ-BR exhibit high reliability and validity and are among the most-commonly used metrics in this research area (5, 8, 9).

Using data from the RMET, AQ, and SPQ, we tested two specific hypotheses. First, we hypothesized that RMET performance should be most directly associated with social abilities and interests, given that cognitive empathy represents a linchpin of effective social interaction. This hypothesis predicts that RMET should be associated most strongly with lower scores on AQ-Social Skills, AQ-Communication, and SPQ-Interpersonal subscales (SPQ-Social Anxiety and SPQ-Constricted Affect). Second, we hypothesized that RMET performance should be associated with aspects of imagination, given that this task centrally involves intuitive inference and conjecturing of the mental states and emotions of others. This hypothesis predicts that RMET performance should be associated most strongly with AQ-Imagination, SPQ-Magical Ideation, and other subscales of the higher-level scale SPQ-Cognitive-Perceptual, that comprises Ideas of Reference (essentially, paranoia), Unusual Perceptions, and Magical Thinking) and thus represents positive schizotypy. For both hypotheses, we considered the effects of sex differences, given that autism involves male biases [e.g., (8)], and positive schizotypy involves female biases [e.g., (7)]. Note that for AQ-Imagination, higher scores represent worse imagination.

## Methods

The study was approved by the Research Ethics boards of Simon Fraser University (2010s0554) and the University of Alberta (Pro00015728), and all participants gave prior written informed consent. Questionnaire data were collected from 1,168 healthy undergraduate psychology students (719 females and 449 males, mean age 19.4 years, SD 2.8, range 17–54 for females, 19.5, SD 2.3, range 16–41 for males) using pencil and paper. This gender imbalance in the sample sizes resulted in greater statistical power for the analyses that were restricted to females, although the sample size for males was still quite large.

The Schizotypal Personality Questionnaire - Brief Revised (SPQ-BR) (9) comprises 32 items that are divided into seven subscales that include (1) ideas of reference, (2) magical thinking, (3) unusual perceptions, (4) constricted affect, (5) social anxiety, (6) odd speech, and (7) eccentric behavior. Subscales 1–3 comprise the higher level scale Cognitive-Perceptual traits (positive schizotypy), 4–5 represent Interpersonal traits, and 6–7 are Disorganized traits, all summing to a total Schizotypy score.

The Autism Spectrum Quotient (AQ) (8) measures the extent to which individuals endorse questions associated with the autistic spectrum. The questionnaire is comprised of 50 items that assess psychological variation across five domains that include (1) communication, (2) social skills, (3) imagination, (4) attention to detail, and (5) attention switching, summing to a total Autism Spectrum score.

The Reading the Mind in the Eyes Test (5), which as noted above quantifies cognitive empathy and emotion recognition, uses 36 pictures of the eye regions of faces that are each surrounded by four choices for the emotion or mental state portrayed, one of which is correct and is scored as a “1,” while incorrect replies are scored as “0.” The 36 pictures were classified into positive, negative, and neutral mental states, using the classification developed by Harkness et al. (10), to assess the possible effects of emotionality cues on RMET performance (for example, “upset” is negative, “friendly” is positive, and “reflective” is neutral) (10).

Analyses were conducted in R v4.0.3 (11). Correlations (Pearson product-moment) of RMET scores with AQ and SPQ subscales were subject to 24-fold Bonferroni adjustments (12 for the subscales, and 2 for males vs. females), yielding a threshold *p*-value of 0.05/24 = 0.0021. Multiple regression analyses were conducted on all main effect terms simultaneously with the base R lm() function; due to the large number of main effects the analyses were conducted without interaction terms. Multiple regression analysis was used to test for the effect of each subscale on RMET performance when holding the values of the other subscales constant statistically, and to test for the level of predictive ability of the full set of independent variables.

## Results

### Sex Differences

Females scored more highly than males on the RMET overall (Table 1). This female advantage was, however, restricted to eyes that showed positive or negative mental states, for which females scored higher than males; for eyes that showed neutral expression, the scores of females and males were not statistically different. Females also scored higher than males for eyes with positive emotions or negative emotions analyzed separately (Table 1). AQ scores were significantly male biased for the AQ-Imagination subscale, and SPQ scores were female-biased for the SPQ-Magical Thinking subscale and male-biased for the SPQ-Constricted Affect subscale (Table 2).

**Table 1:** Sex differences in RMET Total scores by valence of questions.

		**MEAN ± SD (*N*)**	**Males vs. Females Student's** ***t*****-test**		**Effect size**
			***t*-value**	***p*-value**	**(Cohen's *d*)**
RMET Total	♂	25.8 ±4.8 (449)	−4.222	***2.664E-05***	0.26
	♀	27.0 ±4.4 (719)			
RMET Positive	♂	5.6 ±1.6 (449)	−4.295	***1.939E-05***	0.26
	♀	6.0 ±1.5 (719)			
RMET Negative	♂	8.6 ±2.0 (449)	−4.282	***2.045E-05***	0.26
	♀	9.1 ±1.9 (719)			
RMET Neutral	♂	11.6 ±2.6 (449)	−1.750	0.0805	0.12
	♀	11.9 ±2.5 (719)			
RMET Positive + Negative	♂	14.2 ±2.9 (449)	−5.413	***8.001E-08***	0.33
	♀	15.1 ±2.6 (719)			

*Boldface italicized shows Bonferroni-adjusted significance*.

**Table 2:** Sex differences in AQ and SPQ-BR.

**AQ and SPQ scales**		**MEAN ± SD (*N*)**	**Males vs. Females Student's** ***t*****-test**		**Effect size**
			***t*-value**	***p*-value**	**(Cohen's *d*)**
AQ-Social Skills	♂	2.5 ± 2.2 (449)	−1.190	0.234	0.05
	♀	2.6 ± 2.2 (719)			
AQ-Attention Switching	♂	5.0 ± 1.9 (449)	−1.464	0.144	0.10
	♀	5.2 ± 2.0 (719)			
AQ-Attention to Detail	♂	5.5 ± 2.1 (449)	−1.301	0.194	0.10
	♀	5.7 ± 2.1 (719)			
AQ-Communications	♂	2.6 ± 1.9 (449)	−0.045	0.964	0
	♀	2.6 ± 1.9 (719)			
AQ-Imagination	♂	2.8 ± 1.9 (449)	4.535	***6.59E-06***	0.28
	♀	2.3 ± 1.6 (719)			
AQ-TOTAL	♂	18.4 ± 5.8 (449)	−0.047	0.962	0.02
	♀	18.5 ± 5.7 (719)			
SPQ-Ideas of Reference	♂	17.6 ± 4.2 (449)	−2.123	0.034	0.12
	♀	18.1 ± 4.1 (719)			
SPQ-Constricted Affect	♂	16.4 ± 5.0 (449)	2.716	**6.74E-03**	0.18
	♀	15.5 ± 5.0 (719)			
SPQ-Eccentric Behavior	♂	12.4 ± 3.7 (449)	3.659	**2.67E-04**	0.22
	♀	11.6 ± 3.7 (719)			
SPQ-Social Anxiety	♂	11.7 ± 3.7 (449)	−2.291	**0.022**	0.13
	♀	12.2 ± 4.0 (719)			
SPQ-Magical Thinking	♂	7.8 ± 3.4 (449)	−6.061	***1.89E-09***	0.36
	♀	9.1 ± 3.8 (719)			
SPQ-Odd Speech	♂	13.3 ± 2.9 (449)	−3.241	**1.23E-03**	0.21
	♀	13.9 ± 2.9 (719)			
SPQ-Unusual Perception	♂	10.8 ± 2.6 (449)	1.987	**0.047**	0.11
	♀	10.5 ± 2.9 (719)			
SPQ-TOTAL	♂	90.0 ± 14.7 (449)	−1.041	0.298	0.11
	♀	91.0 ± 15.7 (719)			

*Boldface shows nominal significance, and boldface italicized shows Bonferroni-adjusted significance*.

### Correlations of RMET With AQ and SPQ-BR Scales

AQ scores were significantly negatively correlated with RMET Total for the AQ-Communication subscale in males, and for the AQ-Imagination subscale in both sexes (Table 3). SPQ scores were significantly negatively correlated with RMET Total for the SPQ-Ideas of Reference subscale in females, for the SPQ-Magical Thinking subscale in both sexes, and for SPQ-Total in females. The correlations of male and female subscale scores with RMET scores differed only slightly between RMET positive, negative, and neutral questions, for almost all of the tests (average range from lowest to highest of 0.06 correlation coefficient units across both sexes and all subscales, with the largest range for SQP-Magical Thinking in females, of −0.04, −0.20, and −0.21 for positive, negative, and neutral questions).

**Table 3:** Pearson product-moment correlations of RMET Total with AQ and SPQ-BR scales.

**Correlations of RMET Total with AQ and SPQ scales**	**Sex**	**Pearson correlations**	
		***r-*value**	***p*-value**
AQ-Social Skills	♂	−0.0449	0.3427
	♀	−0.0703	0.0596
AQ-Attention Switching	♂	−0.0082	0.8616
	♀	−0.0303	0.4177
AQ-Attention to Detail	♂	−0.0033	0.9450
	♀	0.0519	0.1645
AQ-Communication	♂	−0.1737	***2.161E-04***
	♀	−0.0956	**0.0103**
AQ-Imagination	♂	−0.1725	***2.403E-04***
	♀	−0.1677	***6.139E-06***
AQ-Total	♂	−0.1332	**0.0047**
	♀	−0.0986	**0.0082**
SPQ-Ideas of Reference	♂	−0.1113	**0.0183**
	♀	−0.1906	***2.621E-07***
SPQ-Constricted Affect	♂	−0.0965	**0.0410**
	♀	−0.1020	**0.0062**
SPQ-Eccentric Behavior	♂	−0.0145	0.7597
	♀	0.0402	0.2814
SPQ-Social Anxiety	♂	−0.0022	0.9636
	♀	−0.0333	0.3720
SPQ-Magical Thinking	♂	−0.1728	***2.343E-04***
	♀	−0.2190	***2.938E-09***
SPQ-Odd Speech	♂	−0.0452	0.3397
	♀	−0.0080	0.8305
SPQ-Unusual Perceptions	♂	−0.1387	**0.0032**
	♀	−0.1081	**0.0037**
SPQ-Total	♂	−0.1417	**0.0026**
	♀	−0.1550	***2.976E-05***

*Boldface shows nominal significance, and boldface italicized shows Bonferroni-adjusted significance*.

### Multiple Regression Analysis

Multiple regression analysis yielded an overall highly significant result (*F* = 7.97, df = 13,1154, *p* = 1.96 × 10^−15^, multiple *R*^2^ = 0.082). There was a highly significant effect of sex, and strongest partial regression coefficients were for AQ-Imagination and SPQ-Magical Thinking, both of them negative in direction (Table 4). Surprisingly, positive partial multiple regression coefficients (one significant, and one marginally non-significant) were returned for AQ-Attention Switching and AQ-Attention to Detail, indicating that higher scores for these subscales predicted higher RMET scores. Weakly significant coefficients were also found for AQ-Communication, and SPQ-Odd Speech, both negative in direction.

**Table 4:** Results from multiple regression analysis of RMET Total score on sex and the 12 AQ and SPQ subscales.

**Independent variables**	**β**	**SE**	***t*-value**	***p*-value**
Sex	−1.0323	0.2765	−3.733	**0.0002**
AQ-Social Skills	0.0934	0.077	1.2055	0.2282
AQ-Attention Switching	0.1469	0.0741	1.9827	**0.0476**
AQ-Attention to Detail	0.1214	0.0635	1.9097	0.0564
AQ-Communication	−0.233	0.0928	−2.51	**0.0120**
AQ-Imagination	−0.445	0.0788	−5.647	** <0.0001**
SPQ-Ideas of Reference	0.1021	0.0730	1.397	0.1624
SPQ-Constricted Affect	−0.127	0.0674	−1.884	0.0597
SPQ-Eccentric Behavior	−0.051	0.0648	−0.791	0.4290
SPQ-Social Anxiety	−0.036	0.0743	−0.486	0.626
SPQ-Magical Thinking	−0.183	0.0513	−3.58	**0.0003**
SPQ-Odd Speech	−0.1326	0.0663	−2.000	**0.0457**
SPQ-Unusual Perception	0.1238	0.0686	1.8031	0.0716

*Significant results are shown in boldface*.

## Discussion

In this study, we used the subscales of the AQ and the SPQ-BR, in a very large data set, to test the hypotheses that RMET performance is associated most strongly with either social abilities and interests (reflected in the AQ-Social and SPQ-Interpersonal subscales) or aspects of imagination (as especially reflected in AQ-Imagination subscale, the SPQ-Magical Thinking subscale and the SPQ-Cognitive-Perceptual subscales more generally). These analyses took account of sex, given the known effects of sex differences with regard to autism, schizotypy and the RMET.

Our main findings were 3-fold. First, we found that females performed better than males overall on the RMET, and for the photographs that displayed eyes with negatively or positively valenced mental states. An overall female advantage has been reported in previous work on the RMET (12), and the lack of a significant advantage for neutral items in our results suggests that this advantage stems in part from better recognition of emotional rather than non-emotional states.

Second, in support of the first hypothesis, scores on the RMET were significantly associated with aspects of imagination. In particular, a lower RMET score was highly significantly associated, in both sexes, with (a) higher scores on AQ-Imagination, which denote an under-expressed social imagination, and with (b) higher scores on SPQ-Magical Thinking and SPQ-Ideas of Reference (in females), which can be considered as reflecting, in part, an over-expressed imagination.

Especially strong associations of RMET scores with AQ-Imagination and SPQ-Magical Thinking were detected in a multiple regression analysis that, using all 12 of the AQ and SPQ subscales, adjusted for the full set of independent variables in computing the coefficients. Intriguingly, this analysis suggested, in addition, that higher scores on the two AQ subscales that quantify aspects of attention, AQ-Attention Switching and AQ-Attention to Detail, may be weakly associated with higher scores on the RMET (with a significant coefficient for AQ-Attention Switching, and a marginally non-significant coefficient for AQ-Attention to Detail). High AQ-Attention Switching reflects more highly focused attention, which in many subjects with clinically diagnosed autism becomes over-focused to a problematic degree (13). AQ-Attention to Detail, in turn, reflects a cognitive style that is highly focused on specific, small-scale, aspects of the environment, especially those that comprise parts of integrated wholes (14). As described in more detail below, more highly focused and detail-oriented attention may contribute to RMET performance, in non-clinical subjects and in clinical subjects who are not subject to large cognitive deficits, through enhanced attention and better detection of subtle visual eye-region cues of mental states and emotions. These results also suggest that high performance in some cognitive tasks can be achieved through a combination of autism-related traits and schizotypy-related traits, as found in a number of previous reports (15).

The alternative, though not exclusive, hypothesis addressed here, that RMET performance was mediated by social skills and interests, was not nearly as strongly supported, given that the associations of AQ-Social and SPQ-Interpersonal subscales (SPQ-Social Anxiety and SPQ-Constricted Affect) with RMET scores were relatively low and not statistically significant. The significant associations of higher AQ-Communication scores with worse RMET performance, in males (in the univariate analysis) and in the multiple regression analysis, do however, suggest some contribution of social-communicative skills to these effects.

Third, the psychiatric correlates of RMET performance detected here are strongly associated with sex biases in the subscales. Thus, AQ-Imagination is consistently the most male-biased of all AQ subscales (16), and SPQ-Magical Thinking is consistently the most female-biased of all schizotypal or schizophrenia spectrum traits (17, 18). This pattern suggests the hypothesis that, with regard to RMET performance, males are relatively prone to errors of under-mentalizing due to a less developed social imagination (as in autism), and females are relatively prone to over-mentalizing due to a more highly developed social imagination (as in positive schizotypy). This hypothesis is consistent with the strong male bias in autism, which most commonly involves under-mentalizing, and the strong female bias in borderline personality disorder, which is the disorder most-directly linked with over-mentalizing (19–21). More over-mentalizing errors in females than males may also result, in part, from an increased level of mistaken interpretations of neutral expressions as emotional ones in females (in accordance with the lack of female advantage only for neutral items), although robust interpretation of this finding requires a more fine-grained analysis of the patterns of errors made by individuals of each sex. The hypothesis that males tend to under-mentalize more, and females tend to over-mentalize more, can be evaluated more directly using a test such as the Movie for the Assessment of Social Cognition (22), which allows direct quantification of different types of mentalizing errors, and using a non-clinical population that is not subject to the pronounced psychological and neurological heterogeneity found in most populations with DSM-V diagnoses.

The findings and inferences described here can usefully be related to other studies on the psychological and psychiatric correlates of variation in RMET performance. The main large-scale correlates of better RMET scores include female sex, better verbal abilities, and, in some studies, measures of higher general intelligence (12, 23, 24). RMET performance reductions have been reported in almost all major psychiatric conditions analyzed to date, with the notable exception of borderline personality disorder, for which subjects show comparable scores to controls overall, in meta-analysis (21). Moreover, psychiatric disorders showing more male-biased overall sex ratios (such as autism and schizophrenia) exhibit greater RMET reductions in patients vs. matched controls than do disorders with more female-biased sex ratios (such as depression, borderline personality, and anorexia) (21). These findings indicate that being male, or being subject to a male-based disorder, is associated with reduced RMET performance. These findings fit with our results as regards female superiority in the RMET overall, and with regard to the relatively strong negative correlation of the male-biased AQ-Imagination subscale with RMET scores.

RMET performance enhancements provide especially useful information about this test because their causes are probably not confounded with sex-related or disorder-related cognitive deficits or reductions in ability. Such enhancements have been found in an intriguing suite of studies, with higher RMET scores being reported in: (a) women with an anxious attachment style (25, 26), a condition that is itself female biased (27); (b) women, but not men, with higher levels of social anxiety (28); (c) women with past major depression, dysphoria, or a maternal history of depression, though not with clinical depression (10, 21, 29–31); (d) women with anorexia nervosa, for emotional RMET cues but not overall (32); (e) women with borderline personality disorder, for emotional RMET cues, or overall, and non-clinical women high in borderline traits (for negative cues only) (17, 33–35); (f) typical males and females who read more literary fiction (36); (g) typical males and females who exhibit higher mindfulness or undergo mindfulness training prior to testing (37–40); and (h) typical males and females who have been administered oxytocin (better scores), MDMA (better scores), or testosterone [worse scores, contingent upon their 24D digit ratios (41–43)].

We propose a simple model to help explain this set of findings, whereby RMET performance is enhanced by high social attention and high but non-pathological levels of imagination. By this model, anxious attachment, high social anxiety, mild depression, anorexia, and borderline personality all involve especially high sensitivity to social-emotional cues and signals from others, that derive predominantly from fear of negative or anxiogenic social appraisals or interactions (21, 44). This sensitivity results from high social motivation, and fosters increased attention to social cues, especially cues related to social emotionality. Associations of RMET with literary fiction and mindfulness may reflect, in part, manifestations of increased positive (rather than negative) social attention, with literary fiction also closely linked with social imagination, and mindfulness associated with enhancements to focused attention that commonly involve prosocial emotionality (45, 46). Finally, oxytocin and MDMA administration have also both been demonstrated to increase attention to positive social-emotional stimuli, whereas testosterone reduces it (47–49); oxytocin and MDMA have also been shown to enhance aspects of imagination or creativity (50, 51).

By the model proposed here, high (but not too high) imagination promotes higher RMET performance because the task centers on inventive, conjectural inferences concerning the mental states of others (52, 53). Mechanistically, the model conceives of enhanced RMET performance as involving a high level of social attention as a precondition, and a high but not excessive level of imagination because reading emotions and mental states requires an intuitive inference. Thus, too low a level of imagination results in no clear mental-state hypothesis being intuitively generated (as in autism), and too high a level produces a hypothesis departing too far from the visible information, and produced more from self-generated than externally-cue-generated cognitive-emotional states (as in psychosis, in the extreme). The idea that cognitive empathy performance depends on imagination of mental states and emotions is also supported by fMRI data showing overlap, within the default mode system, between the neural systems that subserve RMET and those that underlie empathy, theory of mind, social cognition, and imagination, especially with regard to activation patterns and functions of the medial pre-frontal and posterior cingulate cortex (16, 54, 55).

The empirical results described here are compatible with the social attention/optimal imagination model in that the AQ and SPQ subscales that reflect imagination, and more-focused and detail-oriented attention, are related to RMET performance, most clearly and simply from the multiple regression. The primary evidence incompatible with the model is that SPQ-Social Anxiety is not associated with RMET performance, which may be some function of this subscale reflecting general fear of all social interactions, rather than anxiety concerning social appraisal and judgement as characterized, for example, by BPD and anorexia (21, 44).

The main limitations of this article are its use of a student population, which limits generality, the gender imbalance, which produces lower statistical power for males than for females, and the low magnitudes of the correlations of the AQ and SPQ subscales with RMET performance, which are indicative of a low proportion of variance accounted for. That said, the multiple regression analysis *R*^2^ did account for about 8% of the variation overall, and the large sample sizes allowed for detection of statistical significance in tests that could otherwise not reject the null hypotheses.

The main implication of these results for future empirical work is that they should motivate direct tests of the proposed model for RMET performance based on social attention and optimal levels of imagination. More generally, the findings suggest that cognitive empathy has deep roots in imagination. As a result, studies of mental disorders that use RMET, and other tests of cognitive empathy, can benefit from conceptualizing and investigating the connections of empathy with imagination, especially with regard to the causes and consequences of especially high, compared to especially low, levels of mentalizing.

## Data Availability Statement

The raw data supporting the conclusions of this article will be made available by the authors, without undue reservation.

## Ethics Statement

The studies involving human participants were reviewed and approved by the Department of Research Ethics boards of Simon Fraser University (2010s0554) and the University of Alberta (Pro00015728), and all participants gave prior written informed consent. The patients/participants provided their written informed consent to participate in this study.

## Author Contributions

BC conceived, read, wrote, and edited the paper. PH, SR, and PN collected the data. All authors analyzed the data, contributed to the article, and approved the submitted version.

## Conflict of Interest

The authors declare that the research was conducted in the absence of any commercial or financial relationships that could be construed as a potential conflict of interest.
